# The risk of fall-related hospitalisations at entry into permanent residential aged care

**DOI:** 10.1186/s12877-021-02640-w

**Published:** 2021-12-07

**Authors:** Maria C. Inacio, Max Moldovan, Craig Whitehead, Janet K. Sluggett, Maria Crotty, Megan Corlis, Renuka Visvanathan, Steve Wesselingh, Gillian E. Caughey

**Affiliations:** 1 Registry of Senior Australians, South Australian Health and Medical Research Institute, PO Box 11060, Adelaide, SA 5001 Australia; 2 UniSA Allied Health and Human Performance, University of South Australia, Adelaide, SA Australia; 3 College of Medicine and Public Health, Flinders University, Adelaide, SA Australia; 4 Southern Adelaide Local Health Network, SA Health, Adelaide, SA Australia; 5 Centre for Medicine Use and Safety, Faculty of Pharmacy and Pharmaceutical Sciences, Monash University, Parkville, VIC Australia; 6 UniSA Clinical & Health Sciences, University of South Australia, Adelaide, SA Australia; 7 National Health and Medical Research Council, Centre of Research Excellence Frailty Trans-Disciplinary Research to Achieve Healthy Ageing, Adelaide, SA Australia; 8 Adelaide Geriatrics Training and Research with Aged Care Centre (GTRAC), University of Adelaide, Adelaide, SA Australia; 9 Aged and Extended Care Services, Central Adelaide Local Health Network, SA Health, Adelaide, SA Australia; 10 Adelaide Medical School, University of Adelaide, Adelaide, SA Australia

**Keywords:** Falls, Injury, Aged care, Risk-prediction

## Abstract

**Background:**

Entering permanent residential aged care (PRAC) is a vulnerable time for individuals. While falls risk assessment tools exist, these have not leveraged routinely collected and integrated information from the Australian aged and health care sectors. Our study examined individual*,* system, medication, and health care related factors at PRAC entry that are predictors of fall-related hospitalisations and developed a risk assessment tool using integrated aged and health care data.

**Methods:**

A retrospective cohort study was conducted on *N* = 32,316 individuals ≥65 years old who entered a PRAC facility (01/01/2009-31/12/2016). Fall-related hospitalisations within 90 or 365 days were the outcomes of interest. Individual, system, medication, and health care-related factors were examined as predictors. Risk prediction models were developed using elastic nets penalised regression and Fine and Gray models. Area under the receiver operating characteristics curve (AUC) assessed model discrimination.

**Results:**

64.2% (*N* = 20,757) of the cohort were women and the median age was 85 years old (interquartile range 80-89). After PRAC entry, 3.7% (*N* = 1209) had a fall-related hospitalisation within 90 days and 9.8% (*N* = 3156) within 365 days. Twenty variables contributed to fall-related hospitalisation prediction within 90 days and the strongest predictors included fracture history (sub-distribution hazard ratio (sHR) = 1.87, 95% confidence interval (CI) 1.63-2.15), falls history (sHR = 1.41, 95%CI 1.21-2.15), and dementia (sHR = 1.39, 95%CI 1.22-1.57). Twenty-seven predictors of fall-related hospitalisation within 365 days were identified, the strongest predictors included dementia (sHR = 1.36, 95%CI 1.24-1.50), history of falls (sHR = 1.30, 95%CI 1.20-1.42) and fractures (sHR = 1.28, 95%CI 1.15-1.41). The risk prediction models had an AUC of 0.71 (95%CI 0.68-0.74) for fall-related hospitalisations within 90 days and 0.64 (95%CI 0.62-0.67) for within 365 days.

**Conclusion:**

Routinely collected aged and health care data, when integrated at a clear point of action such as entry into PRAC, can identify residents at risk of fall-related hospitalisations, providing an opportunity for better targeting risk mitigation strategies.

**Supplementary Information:**

The online version contains supplementary material available at 10.1186/s12877-021-02640-w.

## Background

Globally, over 37 million falls require medical attention each year, with at-least 165,000 injury related hospitalisations due to falls occurring in Australia alone [1–3]. While falls can be prevented, they continue to be a leading cause of reduced quality of life, need for aged care services, hospitalised injury, and injury-related deaths for older people [4–7]. Health care costs from fall-related injuries in Australia in 2015-16 were estimated to be $3.6 billion, 41% of all spending on injuries [8].

Individuals living in permanent residential aged care (PRAC) facilities (nursing homes or long-term care facilities) are most vulnerable to falls given their age, high burden of frailty, high prevalence of dementia, and use of psychotropic medications and medications that can cause orthostatic hypotension, among other contributing factors [9, 10]. A prospective cohort study in six Australian PRAC facilities reported that 27% of hospitalisations over 12-months were fall injury related [11]. In order to implement evidence-based interventions for falls prevention in PRAC settings, adequate risk assessment, which incorporates identifying high risk individuals and potentially modifiable risk factors, must be employed [12]. While several risk assessment tools or prognostic models for falls risk identification have been developed for use in hospitals, community, or PRAC facilities [13–15], these tools have not leveraged the comprehensive information routinely collected in assessments and administrative records from the Australian aged care and health care sectors. Additionally, these tools have not examined the risk profile of individuals at key periods in their aged care journey, including first entry into PRAC. This may be a particularly vulnerable period in terms of falls risk, with residents unfamiliar with their new surroundings, together with considerable changes to medication regimen and care [16].

In 2017, the Registry of Senior Australians (ROSA) established the integration of the aged care and health care sectors’ information for older Australians who have accessed aged care services, so the experience of individuals navigating these sectors can be understood [17]. The ROSA contains a population-based cohort of people using aged care services (2.9 million individuals) and has developed internationally agreed upon indicators of quality and safety for aged care settings, which includes a fall-related hospitalisation indicator [18]. An examination of the 2016 cohort in residential aged care in Australia showed that 10.1% of South Australian residents had at least one fall that required hospitalisation or emergency department (ED) presentation and 3.3% of facilities had higher than expected incidence of fall-related hospitalisations that year [18].

Using the comprehensive information that comprises ROSA our study aimed to: (1) examine individual*,* system, medication, and health care related factors at PRAC entry that are predictors of fall-related hospitalisations; (2) develop a fall-related hospitalisation risk assessment tool using integrated Australian aged care and health care data; (3) compare the newly developed fall-related hospitalisations risk assessment tool’s performance to an existing falls-risk tool (i.e. Fracture Risk Assessment Tool for Community Dwelling older People (FRAT-up)) [19].

## Methods

### Study design, setting, data source

A retrospective cohort study was conducted using the ROSA [17]. This national dataset contains information on all individuals who have undergone an aged care eligibility assessment and accessed services that require this approval, namely permanent residential aged care, home care packages, transition care, and respite care. ROSA has established the linkage of the national aged care datasets from the Australian Institute of Health and Welfare (AIHW) National Aged Care Data Clearinghouse (NACDC), to the health care datasets from the Australian Government, including the Medicare Benefits Schedule (MBS) and Pharmaceutical Benefits Scheme (PBS), and state health authorities’ hospitalisation data collections.

The specific datasets used in this study from the NACDC include the Aged Care Assessment Program (ACAP), Aged Care Funding Instrument (ACFI), episodes of Residential Aged Care Services, and National Death Index (NDI) [17]. The ACAP dataset provides information on the assessor, person seeking services, and recommended services at the time of eligibiligy assessment [20]. The ACFI dataset provides information on needs assessment performed at entry into PRAC. The episodes of Residential Aged Care Services dataset provides the services and dates they were received. The NDI dataset provides date and cause of death, coded using the International Statistical Classification of Diseases and Related Health Problems, Tenth Revision (ICD-10). The MBS dataset provides information on Australian Government subsidised health care services. The PBS dataset provides information on medications dispensed under the PBS (Australia’s national prescription subsidy scheme), coded using the World Health Organisation Anatomical, Therapeutic and Chemical (ATC) classification [21]. The admitted patient datasets provide inpatient hospital encounters and the ED datasets include ED presentations, both are coded (or mapped) using ICD-10 Australian Modification (ICD-10-AM).

### Study cohort

The study cohort included non-Indigenous individuals ≥65 years old who entered PRAC in a South Australian facility between 01/01/2009 and 31/12/2016, who were not Department of Veterans’ Affairs concession card holders (*N* = 32,316).

### Outcome of interest

Time to the first fall-related hospitalisation, defined as the first fall that resulted in hospitalisation or ED presentation, within 90 days or 365 days after entry into PRAC, were the outcomes of interest. Falls-related hospitalisations were determined using the ‘external cause’ code of the admitted data collection dataset for public hospitals and the ‘diagnosis code’ of the ED datasets using the ICD-10-AM codes listed on Supplementary Table 1. Falls with onset in hospital were not included. To ensure the cohort had a minimum follow-up of 365 days, the follow-up period was 01/01/2009 to 31/12/2017.

### Potential predictors of interest

Individual factors (Table 1, Supplementary Tables 2 and 3) were determined at the aged care eligibility or entry into PRAC assessments and included: age, sex, preferred language (English vs. other), partner/marital status, Socio-economic Indexes for Areas’ (SEIFA) relative socio-economic disadvantage index, SEIFA education and occupation index*,* and SEIFA economic resources index [22], weighted frailty index score [23], health conditions, functional limitations, and needs assessment regarding activities of daily living, behaviour, and complex health care needs at entry into care [24]. SEIFA 2016 was applied by matching the PRAC facility post-code to the SEIFA postal areas. Health conditions were ascertained from the aged care eligibligy assessment and entry into care assessment, where they are coded using the same four digit coding scheme [25]. For certain conditions, including diabetes, cerebrovascular disease, cardiovascular disease, and cancer, several conditions that may have fallen under that group (e.g. various types of cancer) were combined into one group. For the condition of dementia both assessments and the medication-based comorbidity index Rx-Risk-V indicator for dementia, which is based on medications used for treatment of dementia or its symptoms, in the 6 months prior to PRAC entry, were used [26].

**Table 1:** Highlights of *individual and clinical* characteristics at entry into permanent residential aged care, by fall-related hospitalisations within 90 days or 365 days of entry

Characteristics	Total	Fall within 90 days	No fall within 90 days	Fall within 365 days	No fall within 365 days
**Total, N(%)**	32,316	1209(3.7)	31,107(96.3)	3156(9.8)	29,160(90.2)
**Median Age (IQR), years**	85(80-89)	85(81-89)	85(80-89)	85(81-89)	84(79-89)
	**N(%)**	**N(%)**	**N(%)**	**N(%)**	**N(%)**
**Sex**					
Women	20,757(64.2)	745(3.6)	20,012(96.4)	2083(10.0)	18,674(90.0)
Men	11,559(35.8)	464(4.0)	11,095(96.0)	1073(9.3)	10,486(90.7)
**Facility Remoteness (ARIA)**					
Major cities	24,799(76.7)	957(3.9)	23,842(96.1)	2514(10.1)	22,285(89.9)
Inner regional	4057(12.6)	117(2.9)	3940(97.1)	321(7.9)	3736(92.1)
Outer regional	3120(9.7)	118(3.8)	3002(96.2)	290(9.3)	2830(90.7)
Remote or very remote	340(1.1)	17(5.0)	323(95.0)	31(9.1)	309(90.9)
**Health Conditions**^**b**^					
Dementia	16,334(50.5)	734(4.5)	15,600(95.5)	1898(11.6)	14,436(88.4)
Delirium	1286(4.0)	81(6.3)	1205(93.7)	166(12.9)	1120(87.1)
History of fall	6938(21.5)	360(5.2)	6578(94.8)	867(12.5)	6071(87.5)
History of fracture	5244(16.2)	335(6.4)	4909(93.6)	667(12.7)	4577(87.3)
Osteoporosis	6546(20.3)	270(4.1)	6276(95.9)	740(11.3)	5806(88.7)
**Rx-Risk-V Co-morbidity Category**^**a**^					
0–1	1875(5.8)	47(2.5)	1828(97.5)	152(8.1)	1723(91.9)
2–3	5952(18.4)	182(3.1)	5770(96.9)	529(8.9)	5423(91.1)
4–5	8555(26.5)	314(3.7)	8241(96.3)	864(10.1)	7691(89.9)
6–8	10,825(33.5)	448(4.1)	10,377(95.9)	1086(10.0)	9739(90.0)
9+	4627(14.3)	210(4.5)	4417(95.5)	494(10.7)	4133(89.3)
**ROSA Frailty Index Score**^**a**^					
0.00 to 0.09 (least frail)	1086(3.4)	37(3.4)	1049(96.6)	96(8.8)	990(91.2)
0.10 to 0.19	8550(26.5)	273(3.2)	8277(96.8)	764(8.9)	7786(91.1)
0.20 to 0.29	18,173(56.2)	715(3.9)	17,458(96.1)	1834(10.1)	16,339(89.9)
0.3 and over	4442(13.7)	183(4.1)	4259(95.9)	458(10.3)	3984(89.7)
**Number of Unique Medications**					
0–4	3661(11.3)	98(2.7)	3563(97.3)	297(8.1)	3364(91.9)
5–10	11,710(36.2)	405(3.5)	11,305(96.5)	1118(9.5)	10,592(90.5)
11+	16,945(52.4)	706(4.2)	16,239(95.8)	1741(10.3)	15,204(89.7)
**Specific Medications (ATC code)**					
Vitamin K antagonists (B01AA)	4572(14.1)	219(4.8)	4353(95.2)	535(11.7)	4037(88.3)
SSRIs (N06AB)	6744(20.9)	307(4.6)	6437(95.4)	743(11.0)	6001(89.0)
**Activities of Daily Living Level**					
No or minimal impairment	12,021(37.2)	604(5.0)	11,417(95.0)	1155(9.6)	10,866(90.4)
Mild impairment	11,242(34.8)	386(3.4)	10,856(96.6)	1231(11.0)	10,011(89.0)
Moderate impairment	7182(22.2)	144(2.0)	7038(98.0)	608(8.5)	6574(91.5)
Severe impairment	426(1.3)	9(2.1)	417(97.9)	24(5.6)	402(94.4)
**Behavioural Daily Living Level**					
No or minimal impairment	11,506(35.6)	547(4.8)	10,959(95.2)	1314(11.4)	10,192(88.6)
Mild impairment	8682(26.9)	314(3.6)	8368(96.4)	831(9.6)	7851(90.4)
Moderate impairment	7385(22.9)	198(2.7)	7187(97.3)	626(8.5)	6759(91.5)
Severe impairment	3298(10.2)	84(2.5)	3214(97.5)	247(7.5)	3051(92.5)
**Complex Health Care (CHC) Level**					
No CHC needed	13,008(40.3)	583(4.5)	12,425(95.5)	1286(9.9)	11,722(90.1)
1–4 CHC procedures needed	8753(27.1)	316(3.6)	8437(96.4)	944(10.8)	7809(89.2)
5–9 CHC procedures needed	7286(22.5)	208(2.9)	7078(97.1)	660(9.1)	6626(90.9)
≥ 10 CHC procedures needed	1824(5.6)	36(2.0)	1788(98.0)	128(7.0)	1696(93.0)
**Number Unplanned Hospitalisations (year prior)**					
None	12,820(39.7)	367(2.9)	12,453(97.1)	1052(8.2)	11,768(91.8)
1	8661(26.8)	333(3.8)	8328(96.2)	860(9.9)	7801(90.1)
2–4	9194(28.5)	419(4.6)	8775(95.4)	1038(11.3)	8156(88.7)
5+	1641(5.1)	90(5.5)	1551(94.5)	206(12.6)	1435(87.4)
**Number of ED Presentations (year prior)**					
None	11,690(36.2)	339(2.9)	11,351(97.1)	960(8.2)	10,730(91.8)
1	9072(28.1)	310(3.4)	8762(96.6)	837(9.2)	8235(90.8)
2-4	9419(29.1)	453(4.8)	8966(95.2)	1102(11.7)	8317(88.3)
5+	2135(6.6)	107(5.0)	2028(95.0)	257(12.0)	1878(88.0)
**Number of GP Attendances (year prior)**					
0	840(2.6)	13(1.5)	827(98.5)	47(5.6)	793(94.4)
1–5	7768(24.0)	247(3.2)	7521(96.8)	712(9.2)	7056(90.8)
6–15	15,698(48.6)	607(3.9)	15,091(96.1)	1541(9.8)	14,157(90.2)
16+	8010(24.8)	342(4.3)	7668(95.7)	856(10.7)	7154(89.3)

*IQR* Interquartile range, *ARIA* Accessibility/Remoteness Index of Australia, *ROSA* Registry of Senior Australians, *ATC* Anatomical, Therapeutic and Chemical classification codes, *SSRIs* Selective serotonin reuptake inhibitors, *CHC* Complex health care, *PRAC* Permanent residential aged care, *MBS* Medicare Benefits Schedule, *ED* Emergency department, *GP* General practitioner

^a^Missing data N(%):Rx-Risk-V 482(1.5), ROSA Frailty index score 65(0.2)

^b^Conditions were ascertained using the aged care eligibility or entry into care assessments. Dementia was ascertained from the aged care eligibility or entry into care assessments and the dispensing of medications for the treatment of dementia

Medication-related factors (Table 1, Supplementary Table 4) were ascertained from dispensing records in the 90-day period prior to entry into PRAC and included: number of unique medications dispensed (chemical substance ATC 5th level), sedative load rating (i.e. cumulative effect of medications with sedative properties) [27], and medication classes at the chemical subgroup ATC 4th level [21*]*.

System and facility related factors (Table 1, Supplementary Table 2), were ascertained at the time of entry into PRAC and included: provider type (not-for-profit, for-profit, or government) and facility remoteness (based on the Accessibility/Remoteness Index of Australia [28], classified as major cities, inner regional, outer regional, or remote/very remote).

Health care-related factors (Table 1, Supplementary Table 5) were ascertained using the history of hospitalisations in the year prior to entry into PRAC and included: number of hospitalisations (unplanned and potentially preventable hospitalisations [29]), ED presentations (any and potentially preventable ED presentations [29]), and cumulative length of hospital stays. Additional health care-related factors were ascertained using the MBS subsidised health encounters (Table 1, Supplementary Table 6) in the year prior to PRAC entry and included: primary care or specialist attendances (geriatrician, palliative and pain), health assessments, team care arrangements, general practitioner (GP) management plan, and comprehensive medication review.

### Analysis

The cohort and crude outcomes were described using means, standard deviation, medians, interquartile ranges (IQR), frequency and proportions. The study cohort was randomly split into five groups; four (80%, *N* = 25,853) were used for training the models and one (20%, *N* = 6463) was used for testing. An elastic nets penalised regression approach, using a Fine and Gray model with death as a competing risk, was used to select best subsets of variables predicting fall-related hospitalisations. The variables for age and sex (base model) were not penalised, and the year of cohort entry was added when fitting the final models. The predictor variables were selected from consensus among at-least three of four groups that penalised regressions were applied to the cross validation folds of the training samples, and assessed for the top 30 and 40 predictors of 90 days and 365 days falls-related hospitalisation incidence, respectively. To enhance model interpretability and avoid omitted-variable bias, if a single level of a multi-level categorical variable entered the model then the rest of levels were included (excluding the reference).

The proportional hazards assumption was tested using Schoenfeld residuals and its violation assessed from plotting log-log survival with respect to alternative quantiles of linear predictors. Subdistribution hazard ratios (sHR) and 95% confidence intervals (CI) were presented. Each model’s calibration was examined by bootstrapping bias-corrected estimates of predicted vs. observed values based on sub-setting predictions into intervals using ‘calibrate() function’ from R rms package. All calculations used complete-case analysis with < 4.7% of cases excluded due to missing data.

Model discrimination was examined using area under the receiver operating characteritics curve (AUC) [30] from the testing group and an additional out-of-sample validation cohort. This additional *validation cohort* included data from a different Australian state captured in ROSA (New South Wales; NSW). For this validation cohort non-Indigenous individuals ≥65 years old who entered a facility in NSW between 01/01/2009 and 31/12/2016 (*N* = 70,462) were selected. In this cohort, fall-related hospitalisations were ascertained using the first listed ‘external cause’ diagnosis in the admitted data collection (covering public and private hospitals) and ‘diagnosis code’ of the ED datasets using ICD-10-AM codes (Supplementary Table 1).

The externally validated FRAT-up [19] was recreated using the ROSA for this cohort, to compare with our developed fall-related hospitalisations-risk assessment tool’s predictive ability. Supplementary Table 7 shows the coding algorithm to recreate the FRAT-up using our available datasets and Supplementary Table 8 includes the model's sHR estimates using this tool. Discrimination was examined using the AUC in two out-of-sample cohorts as described above.

## Results

### Cohort description

Of the 32,316 individuals studied, 64.2% (*N* = 20,757) were women, the median age was 85 years old (IQR 80-89), 50.5% (*N* = 16,334) had a diagnosis of dementia, and 52.4% (*N* = 16,945) were on 11 or more medications. Almost half (47.8%, *N* = 15,452) of the cohort had six or more comorbid conditions and 21.5% (*N* = 5244) had a history of falls. At entry into PRAC, 23.5% (*N* = 7608) had moderate/high impairments of activities of daily living, 33.1% (*N* = 10,683) had moderate/high daily behavioural needs and 28.1% (*N* = 9110) had 5 or more complex health care needs. Sixty percent (*N* = 19,496) had at-least one unplanned hospitalisation, 63.8% (*N* = 20,626) had at least one ED presentation, and almost all (97.4%, *N* = 31,476) saw a GP in the year prior to PRAC entry. Table 1 includes individual, system and clinical characteristics, Supplementary Tables 2, 3, 4, 5 and 6 includes all other individual, medication and health care-related factors for the study cohort.

After entry into PRAC, 3.7% (*n* = 1209) of residents had a fall-related hospitalisation within 90 days and 9.8% (*n* = 3156) within 365 days (Table 1). The cumulative incidence of mortality was 13.7% (95%CI 13.3-14.0%) within 90 days and 29.2% (95%CI 28.7-29.7%) within 365 days.

### Predictors of fall-related hospitalisations within 90 days of PRAC entry

Twenty variables were contributors to fall-related hospitalisation prediction within 90 days (Table 2). Older individuals (sHR = 1.02, 95%CI 1.02-1.03, per year of increasing age) and men (sHR = 1.16, 95%CI 1.02-1.30) were at a higher risk of fall-related hospitalisations. The strongest predicting factors included history of fractures (sHR = 1.87, 95%CI 1.63-2.15), history of falls (sHR = 1.41, 95% CI 1.21-2.15), dementia (sHR = 1.39, 95%CI 1.22-1.57), and history of delirium (sHR = 1.28, 95%CI 1.02-1.61). In addition, use of a vitamin K antagonist (which includes the oral anticoagulant warfarin, sHR = 1.33, 95%CI 1.12-1.59) or a selective serotonin reuptake inhibitor (SSRI) antidepressant medication (sHR = 1.21, 95%CI 1.03-1.42) were fall-related hospitalisation predictors. Higher numbers of GP attendances in the year prior to entry into PRAC were associated with a dose-dependent increase in the risk of fall-related hospitalisations, with those who saw a GP ≥16 times, having a 3-times higher risk (sHR = 3.31, 95%CI 1.58-6.93) compared to those who did not see a GP. By comparison to individuals with the worst mobility or impaired cognition ratings, those with less severe or no mobility or impaired cognition, respectively, were dose-dependently less likely to have a fall-related hospitalisation (e.g. individuals in the highest mobility, sHR = 0.46, 95%CI 0.24-0.88 or cognitive rating category, sHR = 0.59, 95%CI 0.47-0.74, respectively) (Table 2).

**Table 2:** Predictors of fall-related hospitalisations within 90 days of entry into permanent residential aged care^a^

Variables	sHR	95%CI	***P***-value
**Time trend, years**	1.06	1.03–1.09	< 0.001
**Age (per 1 year increment)**	1.02	1.02–1.03	< 0.001
**Men vs Women**	1.16	1.02–1.30	0.028
**History of Fractures (yes vs no)**	1.87	1.63–2.15	< 0.001
**History of Falls (yes vs no)**	1.41	1.21–1.64	< 0.001
**Dementia (yes vs no)**	1.39	1.22–1.57	< 0.001
**Delirium (yes vs no)**	1.28	1.02–1.61	0.033
**Rx-Risk-V Co-morbidity Category**			
2–3 vs 0–1	1.10	0.82–1.46	0.530
4–5 vs 0–1	1.29	0.97–1.71	0.080
6–8 vs 0–1	1.20	0.89–1.63	0.240
9+ vs 0–1	1.28	0.91–1.79	0.160
**Medications Sedative Load Rating Category**			
1–2 vs 0	0.93	0.75–1.14	0.460
3+ vs 0	1.11	0.95–1.30	0.170
**Vitamin K Antagonists (ATC code B01AA) (yes vs no)**	1.33	1.12–1.59	0.001
**SSRI (ATC code N06AB) (yes vs no)**	1.21	1.03–1.42	0.020
**Activities of Daily Living Level**			
No or minimal vs Severe impairment	2.24	0.76–6.56	0.140
Mild vs Severe impairment	1.19	0.80–1.76	0.400
Moderate vs Severe impairment	1.06	0.88–1.28	0.520
**Behavioural Daily Living Level**			
No or minimal vs Severe impairment	0.91	0.66–1.24	0.530
Mild vs Severe impairment	0.76	0.64–0.90	0.002
Moderate vs Severe impairment	0.88	0.74–1.04	0.120
**Mobility Rating**			
A best vs D worst	0.46	0.24–0.88	0.019
B vs D worst	0.59	0.41–0.86	0.005
C vs D worst	0.67	0.54–0.84	0.001
**Toileting Rating**			
A best vs D worst	0.70	0.42–1.14	0.150
B vs D worst	0.67	0.47–0.97	0.032
C vs D worst	0.97	0.78–1.19	0.740
**Cognitive Rating**			
A best vs D worst	0.59	0.47–0.74	< 0.001
B vs D worst	0.72	0.60–0.86	< 0.001
C vs D worst	0.78	0.68–0.89	< 0.001
**Depression and Dysthymia Rating**			
A best vs D worst	0.97	0.80–1.17	0.760
B vs D worst	1.01	0.82–1.23	0.950
C vs D worst	1.20	0.97–1.49	0.088
**Number of unplanned hospitalisations (year prior)**			
1 vs 0	1.03	0.86–1.25	0.720
2–4 vs 0	1.04	0.86–1.26	0.710
5+ vs 0	1.27	0.97–1.65	0.083
**Number of ED Presentations (year prior)**			
1 vs 0	1.03	0.86–1.24	0.740
2–4 vs 0	1.36	1.11–1.67	0.003
5+ vs 0	1.21	0.90–1.63	0.210
**Number of GP Attendances (year prior)**			
1–5 vs 0	2.34	1.15–4.78	0.019
6–15 vs 0	2.93	1.42–6.03	0.004
16+ vs 0	3.31	1.58–6.93	0.002

*sHR* Subdistribution hazard ratio, *CI* Confidence intervals, *GP* General practitioners, *ATC* Anatomical, Therapeutic and Chemical classification codes, *ED* Emergency department, *MBS* Medicare Benefits Schedule, *SSRI* Selective serotonin reuptake inhibitor

^a^*N* = 30,871 included in models. 1445 (4.5%) of cohort not included due to missing data

### Predictors of fall-related hospitalisations within 365 days of PRAC entry

Twenty-seven variables were predictors of fall-related hospitalisations within 365 days (Table 3). Older individuals were at a higher risk of fall-related hospitalisations (sHR = 1.02, 95%CI 1.02-1.03, per year of increasing age) (Table 3) and the strongest predictors included dementia (sHR = 1.36, 95%CI 1.24-1.50), history of falls (sHR = 1.30, 95% CI 1.20-1.42), history of fractures (sHR = 1.28, 95%CI 1.15-1.41), and osteoporosis (sHR = 1.13, 95%CI 1.03-1.25). In terms of medication use, those dispensed a vitamin K antagonist (sHR = 1.30, 95%CI 1.17-1.45) or a SSRI (sHR = 1.15, 95%CI 1.04-1.27) had a higher risk of having a fall-related hospitalisation. By comparison to individuals with poor nutrition, those with better nutrition ratings had a greater risk of fall-related hospitalisations (e.g. highest level of nutrition rating sHR = 1.57, 95%CI 1.27-1.94). Increasing numbers of unplanned hospitalisations and ED presentations in the year prior to entering PRAC were associated with a higher fall-related hospitalisation risk (e.g. ≥5 unplanned hospitalisations sHR = 1.90, 95%CI 1.51-2.39 and ≥ 5 ED presentations sHR = 1.28, 95%CI 1.06-1.54). Increasing numbers of GP attendances in the year prior to entry were associated with a dose-dependent higher risk of fall-related hospitalisations, with those who had ≥16 GP visits having a 2-fold (sHR = 1.99, 95%CI 1.41-2.83) higher risk compared to those who did not see a GP. Compared to individuals with the lowest level of frailty, those with higher frailty scores had lower risks of fall-related hospitalisations (e.g. frailty index 0.6-0.8 sHR = 0.71, 95%CI 0.57-0.89) (Table 3). Higher cognitive function was associated with a lower risk of fall-related hospitalisation within 365 days (e.g. individuals in the highest cognitive rating category sHR = 0.63, 95%CI 0.53-0.75). The more recent an unplanned hospitalisation to the time of PRAC entry, the less likely individuals were to have a fall-related hospitalisation (e.g. 0-20 days from hospitalisation to entering PRAC sHR = 0.72, 95%CI 0.62-0.84).

**Table 3:** Predictors of fall-related hospitalisations within 365 days of entry into permanent residential aged care^a^

Variables	sHR	95%CI	***P***-value
**Time trend, years**	1.07	1.05–1.09	< 0.001
**Age (per 1 year increment)**	1.02	1.02–1.03	< 0.001
**Men vs Women**	1.00	0.94–1.08	0.900
**Facility Remoteness**			
Inner Regional vs Major Cities	0.80	0.72–0.91	< 0.001
Outer Regional vs Major Cities	0.93	0.80–1.07	0.300
Remote or Very Remote vs Major Cities	0.91	0.61–1.35	0.640
**History of Fractures (yes vs no)**	1.28	1.15–1.41	< 0.001
**History of Falls (yes vs no)**	1.30	1.20–1.42	< 0.001
**Dementia (yes vs no)**	1.36	1.24–1.50	< 0.001
**Osteoporosis (yes vs no)**	1.13	1.03–1.25	0.013
**Normalised Weighted ROSA Frailty Index Category**			
[0.2–0.4) vs [0–0.2)	0.79	0.65–0.96	0.019
[0.4–0.6) vs [0–0.2)	0.76	0.62–0.93	0.007
[0.6–0.8) vs [0–0.2)	0.71	0.57–0.89	0.003
[0.8–1.0] vs [0–0.2)	0.47	ND	0.870
**Rx-Risk-V Co-morbidity Category**			
2–3 vs 0–1	0.99	0.84–1.17	0.900
4–5 vs 0–1	1.11	0.94–1.30	0.210
6–8 vs 0–1	1.07	0.90–1.28	0.440
9+ vs 0–1	1.14	0.95–1.37	0.170
**Vitamin K Antagonists (ATC Code B01AA) (yes vs no)**	1.30	1.17–1.45	< 0.001
**SSRI (ATC Code N06AB) (yes vs no)**	1.15	1.04–1.27	0.007
**Activities of Daily Living Level**			
No or minimal vs Severe impairment	1.01	0.56–1.83	0.970
Mild vs Severe impairment	0.99	0.80–1.22	0.930
Moderate vs Severe impairment	1.02	0.88–1.18	0.810
**Behavioural Daily Living Level**			
No or minimal vs Severe impairment	0.93	0.78–1.11	0.440
Mild vs Severe impairment	0.89	0.78–1.00	0.059
Moderate vs Severe impairment	0.92	0.84–1.01	0.077
**Complex Health Care (CHC) Needs Level**			
No vs ≥10 CHC procedures needed	1.02	0.87–1.20	0.820
1–4 vs ≥10 CHC procedures needed	1.07	0.97–1.18	0.150
5–9 vs ≥10 CHC procedures needed	1.11	1.01–1.22	0.026
**Nutrition Rating**			
A best vs D worst	1.57	1.27–1.94	< 0.001
B vs D worst	1.46	1.22–1.74	< 0.001
C vs D worst	1.59	1.40–1.81	< 0.001
**Mobility Rating**			
A best vs D worst	0.74	0.53–1.04	0.088
B vs D worst	0.92	0.74–1.14	0.450
C vs D worst	0.99	0.85–1.15	0.880
**Cognitive Rating**			
A best vs D worst	0.63	0.53–0.75	< 0.001
B vs D worst	0.78	0.69–0.88	< 0.001
C vs D worst	0.80	0.71–0.89	< 0.001
**Wandering Rating**			
A best vs D worst	0.88	0.78–1.00	0.053
B vs D worst	1.09	0.93–1.26	0.290
C vs D worst	1.04	0.87–1.23	0.690
**Depression and Dysthymia Rating**			
A best vs D worst	0.92	0.81–1.05	0.200
B vs D worst	0.98	0.86–1.11	0.710
C vs D worst	1.07	0.94–1.23	0.310
**Number of Unplanned Hospitalisations (year prior)**			
1 vs 0	1.30	1.10–1.52	0.002
2–4 vs 0	1.45	1.21–1.75	< 0.001
5+ vs 0	1.90	1.51–2.39	< 0.001
**Number of ED Presentations (year prior)**			
1 vs 0	1.07	0.95–1.20	0.270
2–4 vs 0	1.29	1.13–1.48	< 0.001
5+ vs 0	1.28	1.06–1.54	0.011
**Number of Unplanned Potentially Preventable Hospitalisations (year prior)**		–	–
1 vs 0	0.97	0.87–1.09	0.630
2–4 vs 0	0.85	0.72–1.02	0.082
5+ vs 0	0.23	0.02–2.75	0.250
**Days Between Last Unplanned Hospitalisation and Entry into PRAC **			
0–20 vs 151+	0.72	0.62–0.84	< 0.001
21–100 vs 151+	0.80	0.71–0.90	< 0.001
101–150 vs 151+	0.75	0.63–0.90	0.002
**Number of GP Attendances (year prior)**			
1–5 vs 0	1.59	1.13–2.22	0.007
6–15 vs 0	1.75	1.25–2.45	0.001
16+ vs 0	1.99	1.41–2.83	< 0.001
**Number of GP Management, Multidisciplinary/Team Care Plans (year prior)**			
1 vs 0	1.03	0.91–1.18	0.610
2–4 vs 0	1.00	0.92–1.09	0.980
5+ vs 0	1.04	0.81–1.33	0.770
**Number of 75+ Health Assessments (year prior)**			
1 vs 0	1.08	0.98–1.19	0.130
2+ vs 0	0.99	0.69–1.43	0.970

*sHR* Subdistribution hazard ratio, *CI* Confidence intervals, *ROSA* Registry of Senior Australians, *ATC* Anatomical, Therapeutic and Chemical classification codes, *GP* General practitioners, *ED* Emergency department, *MBS* Medicare Benefits Schedule, *ND* Not defined, confidence interval not defined due to inflated standard error

^a^*N* = 30,808 included in models. 1508 (4.7%) of cohort not included due to missing data

### Model performance (out of sample and compared to FRAT-up)

**Fig. 1:**
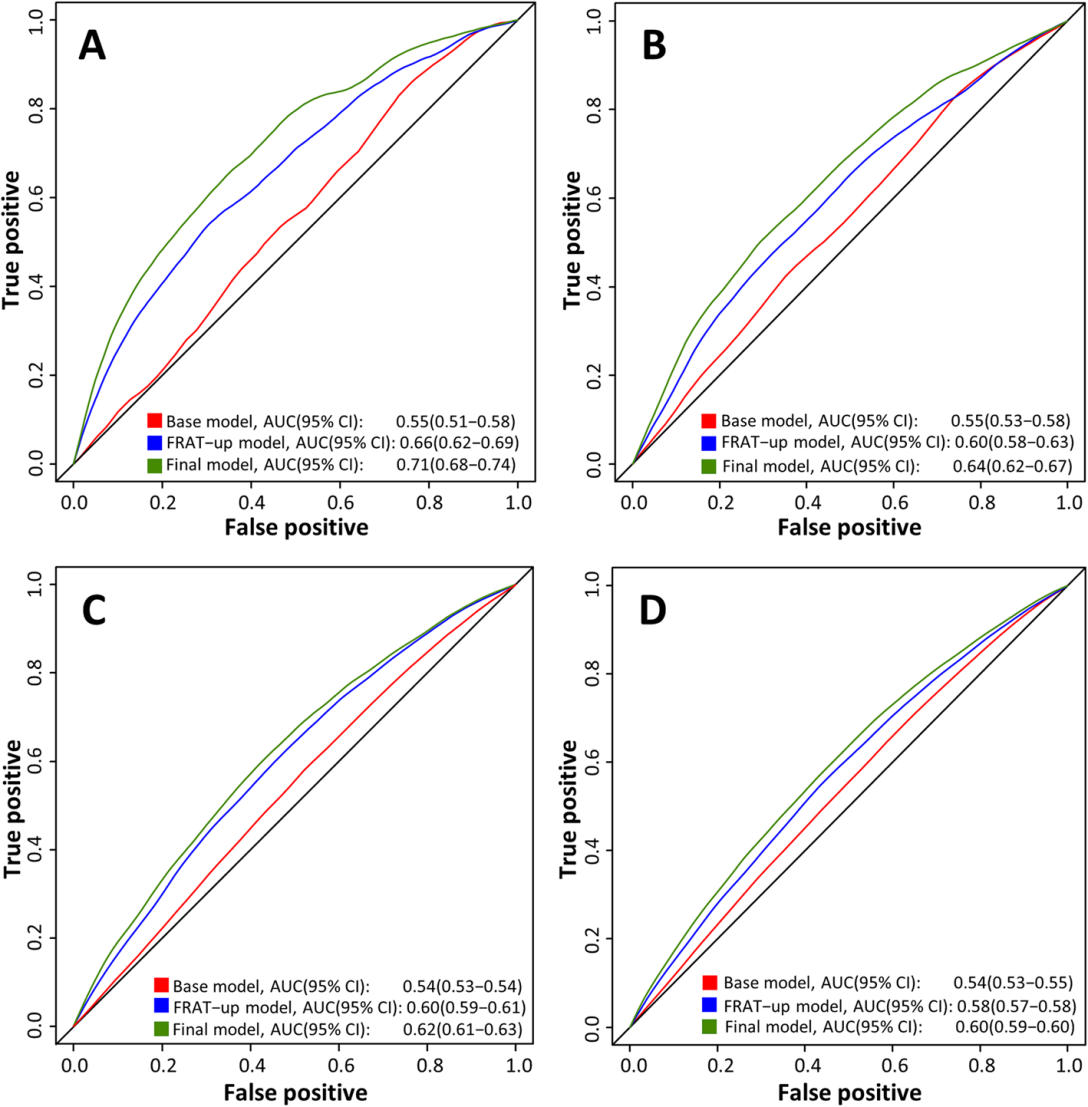
Proposed prognostic model and FRAT-up model performance to estimate risk of fall-related hospitalisations within 90 and 365 days post entry into permanent residential care out of sample, Area Under the Receiver Operating Characteristics Curve (AUC). **A** 20% out-of-sample validation cohort, fall-related hospitalisation within 90 days. **B** 20% out-of-sample validation cohort, fall-related hospitalisation within 365 days. **C** New South Wales validation cohort, fall-related hospitalisation within 90 days. D. South Wales validation cohort, fall-related hospitalisation within 365 days

Validation of the risk prediction model using the 20% testing cohort (*N* = 6463) showed an out-of-sample predictive ability (AUC) of 0.71 (95%CI 0.68-0.74) for fall-related hospitalisation within 90 days and 0.64 (95%CI 0.62-0.67) for fall-related hospitalisation within 365 days (Table 4, Fig. 1). This was higher than when compared to the FRAT-up model for both 90 days (0.66, 95% CI 0.62-0.69) and 365 days (0.60, 95% CI 0.58-0.66). Predictive ability for the model and the FRAT-up model was lower in the NSW out-of-sample validation cohort for 90 days (0.62, 95% CI 0.61-0.63) and 365 days (0.60, 95%CI 0.59-0.61) (Table 4, Fig. 1).

**Table 4:** Proposed prognostic model and FRAT-up model performance to estimate risk of fall-related hospitalisations within 90 and 365 days post entry into permanent residential aged care

	Model performance, AUC (95%CI)^**a**^	
	Out-of-sample validation, SA^**b**^	Out-of-sample validation, NSW^**c**^
**Fall-related hospitalisations within 90 days**		
Proposed Model	0.71(0.68–0.74)	0.62(0.61–0.63)
FRAT-up Model	0.66(0.62–0.69)	0.60(0.59–0.61)
**Fall-related hospitalisations within 365 days**		
Proposed Model	0.64(0.62–0.67)	0.60(0.59–0.60)
FRAT-up Model	0.60(0.58–0.66)	0.58(0.57–0.58)

*AUC* Area under the receiver operating characteritics curve, *CI* Confidence intervals, *SA* South Australia, *NSW* New South Wales

^a^Area under the receiver operating characteristic curve

^b^Validation performed on 20% South Australian cohort not used in models’ development

^c^Validation performed on individuals’ entering residential aged care in New South Wales between 2012 and 2017

## Discussion

A risk prediction model for fall-related hospitalisations for individuals entering PRAC was developed using individual*,* system, medication, and health care related characteristics routinely collected in Australia. Twenty and 27 factors contributing to the risk of fall-related hospitalisation within 90 days and 365 days of entry into PRAC, respectively were identified. With approximately 70,000 individuals entering PRAC each year and assessment of clinical needs occurring during this transition period, this is an important time for clinicians and aged care providers to identify, counsel, and minimise the risk of falls and therefore fall-related hospitalisations. The recent Royal Commission into Aged Care Quality and Safety in Australia highlighted the need for a more resident-centred model of care, greater allied health input into resident care, and widespread integration of electronic care systems in facilities, all of which could contribute to mitigating risk of falls [31]. Additionally, a new national quality indicator to monitor the proportion of residents with a fall and a fall that results in a major injury will be incorporated into Australia’s National Aged Care Mandatory Quality Indicator Program in July 2021 [32]. Risk prediction tools that can be automated and applied to identify those at risk of falls and prompt intervention at PRAC entry are likely to be valued by aged care providers, clinicians, residents and family members.

This study found that 3.7% and 9.8% of residents have at least one fall-related hospitalisation within 90 days and 365 days of PRAC entry, respectively. Importantly, we examined time to fall-related hospitalisation while accounting for the competing risk of mortality [33], and determined the most influential predictors. Our analysis contributes to the existing literature by uniquely determining the predictive ability of models applied to a large cohort of individuals at a specific time period in their aged care journey. Although a significant body of literature surrounds the development of risk profiling tools for falls [13–15, 34], most studies have examined the risk of any fall rather than fall-related hospitalisations, and few have been validated in the PRAC setting. Most existing falls risk profiling tools have achieved low to moderate predictive ability (AUC 0.55-0.65) [14, 34]. Few studies have examined the discrimination ability of more sophisticated analytical approaches for fall risk prediction as utilised in our study. Palumbo et al. [14] used a statistical learning approach for their falls prediction model and found that the simpler FRAT-up tool performed similarly. The discrimination of our 365-day model (0.64) is similar to the predictive ability of prior models, however our 90-day model had better, yet moderate predictive ability (0.71).

Risk factors (i.e. factors associated with the outcome) and/or predictors (i.e. factors that contribute to the prediction of the outcome) of falls among older adults are well documented [9, 10, 35]. In our study, several established factors associated with falls were confirmed, including increasing age, being a man, prior history of falls and fractures, challenges with mobility, and cognitive impairment, including dementia and delirium [9, 10]. We confirmed that use of SSRIs is a fall predictor [9, 10, 35, 36]. Other system related factors determined to be fall predictors included the number of GP encounters and unplanned hospitalisations in the year prior to PRAC entry. These have both consistently identified individuals at high risk of hospitalisation [37] and are likely important indications of recent and significant deterioration in individuals’ health, and increased need for support.

Use of anticoagulants was found to be associated with a higher risk of fall-related hospitalisations. The association of anticoagulants is likely due to the significant concerns for potentially severe adverse events associated with a fall in an older individual such as increased risk of bleeding [38]. In South Australia, paramedics are trained to transfer all residents taking an anticoagulant to hospital post-fall for further investigation to exclude intracranial injury [39]. We also found the poorest ratings of nutrition and frailty had the lowest risks of fall-related hospitalisations, contrary to other studies [40, 41]. These paradoxical findings are likely because we accounted for the competing risk of death in our analysis. For example, 67% of people with the lowest nutrition rating die within a year of PRAC entry compared to only 25% with higher nutrition ratings. Additionally, those with the worst rating in frailty are likely not ambulant.

Our study strengths include the complete capture of individuals who have entered PRAC in South Australia during the study period. This large sample and linkage of routinely collected aged and health care records allowed us to examine a comprehensive number of predictors systematically. We have also importantly accounted for the competing risk of death in our analysis, which is as high as 35% in the first year after entering care [42]. The validation of our models out-of-sample using 20% testing cohort in South Australia and from another Australian state, increases the methodological quality of our assessment. Fall-related hospitalisations as the main outcome is an underestimation of the incidence of falls generally, which has been estimated to be as high as 50% of older people in PRAC facilities [39, 43]. However, it captures significant falls (i.e. resulting in hospitalisation) likely associated with the greatest morbidity, mortality and costs. Our study was also limited to the South Australian cohort, which in 2018 represented 8% of the new entrants to PRAC nationally [20]. Additionally, only fall-related hospitalisations in public hospitals were included, but this captures 92% of unplanned hospitalisations in Australia. Our estimate of frailty is also likely underreported as it is captured at the time of the aged care eligibiligy assessment (median time between eligibiligy assessment and entry into PRAC is 258 days) and individuals’ frailty levels could have worsened [44]. ROSA does not contain some of the in-depth clinical, environmental, and biological level data that may also contribute to the risk of fall-related hospitalisations at a given point in time. For example, we were unable to examine recent medication dose changes or specific combinations of medication use which may impact fall-related hospitalisation risk. Under-ascertainment of conditions and missing data are possible when using existing datasets. However, these potential limitations were mitigated by conducting logic checks within and between datasets and using multiple points of capture to confirm events/conditions, and the authority responsible for the data linkage in ROSA reports high matching rates (> 98%). Similary, the observational nature of our data means we are unable to infer causality from associations. Finally, predictive ability was lower in the validation cohort, which could reflect state differences in how fall-related hospitalisations are coded or facility protocols related to hospital transfers.

## Conclusion

This exhaustive investigation has identified several individual and health care characteristics, medication, and system-related factors that predict fall-related hospitalisations for older Australians at time of entry into PRAC; a clear point of action. Our findings highlight the ability to utilise integrated routinely collected data to identify residents who are at higher risk of fall-related hospitalisations, thereby providing opportunity to better target strategies to minimise falls risk and related harms.

## Supplementary Information

**Additional file 1: Supplementary Table 1.** Hospitalisation coding algorithm to identify fall-related hospitalisations. **Supplementary Table 2. ***Individual characteristics* at entry into permanent residential aged care by fall-related hospitalisations within 90 days or 365 days of entry (not included in Table 1). **Supplementary Table 3. ***Individual characteristics* at entry into permanent residential aged care from Aged Care Funding Instrument Assessment^1^ by fall-related hospitalisations within 90 days or 365 days of entry (not included in Table 1). **Supplementary Table 4. ***Medication use c*haracteristics within 90 days prior to entry into permanent residential aged care by fall-related hospitalisations within 90 days or 365 days of entry (not included in Table 1). **Supplementary Table 5.** Hospital related health care utilisation within 1 year prior of entry into permanent residential aged care by individuals’ fall-related hospitalisations within 90 days or 365 days of entry (not included in Table 1). **Supplementary Table 6. ***Other health care service utilisation* within 1 year prior of entry into permanent residential aged care by individuals’ fall-related hospitalisations within 90 days or 365 days of entry (not included in Table 1). **Supplementary Table 7.** Variables and coding used to recreate risk factors included in the Fracture Risk Assessment Tool for Community Dwelling Older People (FRAT-up)^2^ and prevalence of risk factor in the study cohort. **Supplementary Table 8.** Risk Estimates Using the Fracture Risk Assessment Tool for Community Dwelling Older People (FRAT-up)^2^.

## Data Availability

The data for this study were obtained from the Australian Government Department of Health and South Australia and New South Waltes state health authorities and integrated by the Australian Institute of Health and Welfare, Centre for Health Record Linkage, and SA NT DataLink. These data were made available to the researchers under ethical, governance, and confidentiality agreements that do not allow public sharing.

## Abbreviations

ACAP: Aged Care Assessment Program
ACFI: Aged Care Funding Instrument
AIHW: Australian Institute of Health and Welfare
ATC: Anatomical, Therapeutical and Chemical
CI: Confidence intervals
ED: Emergency department
FRAT: Fracture risk assessment tool
GP: General practitioner
IQR: Interquartile range
MBS: Medicare Benefits Schedule
NACDC: National Aged Care Data Clearinghouse
NDI: National Death Index
NSW: New South Wales
PBS: Pharmaceutical Benefits Scheme
PRAC: Permanent Residential Aged Care
ROSA: Registry of Senior Australians
SEIFA: Socio-economic Indexes for Areas
sHR: Sub-distribution hazard ratio
SSRI: Selective serotonin reuptake inhibitor
