# Supplementary material for: The risk of fall-related hospitalisations at entry into permanent residential aged care
Source: BMC Geriatr. 2021 Dec 7;21:686. doi: 10.1186/s12877-021-02640-w (PMC8650418; doi:10.1186/s12877-021-02640-w)
Supplement: Supplementary file 1 — Additional file 1: Supplementary Table 1. Hospitalisation coding algorithm to identify fall-related hospitalisations. Supplementary Table 2. Individual characteristics at entry into permanent residential aged care by fall-related hospitalisations within 90 days or 365 days of entry (not included in Table 1). Supplementary Table 3. Individual characteristics at entry into permanent residential aged care from Aged Care Funding Instrument Assessment1 by fall-related hospitalisations within 90 days or 365 days of entry (not included in Table 1). Supplementary Table 4. Medication use characteristics within 90 days prior to entry into permanent residential aged care by fall-related hospitalisations within 90 days or 365 days of entry (not included in Table 1). Supplementary Table 5. Hospital related health care utilisation within 1 year prior of entry into permanent residential aged care by individuals’ fall-related hospitalisations within 90 days or 365 days of entry (not included in Table 1). Supplementary Table 6. Other health care service utilisation within 1 year prior of entry into permanent residential aged care by individuals’ fall-related hospitalisations within 90 days or 365 days of entry (not included in Table 1). Supplementary Table 7. Variables and coding used to recreate risk factors included in the Fracture Risk Assessment Tool for Community Dwelling Older People (FRAT-up)2 and prevalence of risk factor in the study cohort. Supplementary Table 8. Risk Estimates Using the Fracture Risk Assessment Tool for Community Dwelling Older People (FRAT-up)2. [file 12877_2021_2640_MOESM1_ESM.docx]

**Supplementary Tables and References**

**Supplementary Table 1. Hospitalisation coding algorithm to identify fall-related hospitalisations**

| **Description** | **ICD-10-AM Codes^1^** |
| --- | --- |
| Fall due to ice and snow | W00* |
| Fall on same level from slipping, tripping and stumbling | W01* |
| Other fall on same level due to collision with another person | W03* |
| Fall while being carried or supported by other persons | W04* |
| Fall from non-moving wheelchair, nonmotorized scooter and motorized mobility scooter | W05* |
| Fall from bed | W06* |
| Fall from chair | W07* |
| Fall from other furniture | W08* |
| Fall on and from playground equipment | W09* |
| Fall on and from stairs and steps | W10* |
| Fall on and from ladder | W11* |
| Fall on and from scaffolding | W12* |
| Fall from, out of or through building or structure | W13* |
| Fall from tree | W14* |
| Fall from cliff | W15* |
| Fall, jump or diving into water | W16* |
| Other fall from one level to another | W17* |
| Other slipping, tripping and stumbling and falls | W18* |
| Unspecified fall | W19* |
| Tendency to fall, not elsewhere classified^2^ | R29.6 |

ICD-10-AM=International Classification of Diseases, 10^th^ Revision, Australian Modification.

1. In Inpatient South Australian Admitted Care (ISAAC) dataset: external cause code. In Non-admitted emergency activity (EDDC) dataset: diagnosis code.

**Supplementary Table 2.** ***Individual characteristics* at entry into permanent residential aged care by fall-related hospitalisations within 90 days or 365 days of entry (not included in Table 1)**

| **Characteristics** | **Total N(%)** | **Fall within 90 days N(%)** | **No fall within 90 days N(%)** | **Fall within 365 days N(%)** | **No fall within 365 days N(%)** |
| --- | --- | --- | --- | --- | --- |
| **Total** | **32316** | **1209(3.7)** | **31107(96.3)** | **3156(9.8)** | **29160(90.2)** |
| **Age group, years** |  |  |  |  |  |
| 65-74 | 3450(10.7) | 72(2.1) | 3378(97.9) | 203(5.9) | 3247(94.1) |
| 75-84 | 12630(39.1) | 479(3.8) | 12151(96.2) | 1177(9.3) | 11453(90.7) |
| 85-94 | 14586(45.1) | 588(4.0) | 13998(96.0) | 1578(10.8) | 13008(89.2) |
| 95-105 | 1650(5.1) | 70(4.2) | 1580(95.8) | 198(12.0) | 1452(88.0) |
| **English the first language^1^** | 28546(88.3) | 1065(3.7) | 27481(96.3) | 2806(9.8) | 25740(90.2) |
| **Born in Australia^1^** | 20185(62.5) | 724(3.6) | 19461(96.4) | 1901(9.4) | 18284(90.6) |
| **SEIFA Index of Relative Socio-economic Disadvantage^1^** |  |  |  |  |  |
| Q1- Greater disadvantage and lack of advantage | 8325(25.8) | 314(3.8) | 8011(96.2) | 790(9.5) | 7535(90.5) |
| Q2 | 5327(16.5) | 193(3.6) | 5134(96.4) | 536(10.1) | 4791(89.9) |
| Q3 | 7685(23.8) | 303(3.9) | 7382(96.1) | 774(10.1) | 6911(89.9) |
| Q4 | 6721(20.8) | 260(3.9) | 6461(96.1) | 666(9.9) | 6055(90.1) |
| Q5- Low disadvantage and greater advantage | 4237(13.1) | 137(3.2) | 4100(96.8) | 387(9.1) | 3850(90.9) |
| **SEIFA Index of Economic Resources^1^** |  |  |  |  |  |
| Q1- Relative lack of access to economic resources | 12376(38.3) | 462(3.7) | 11914(96.3) | 1226(9.9) | 11150(90.1) |
| Q2 | 7616(23.6) | 289(3.8) | 7327(96.2) | 732(9.6) | 6884(90.4) |
| Q3 | 6902(21.4) | 274(4.0) | 6628(96.0) | 667(9.7) | 6235(90.3) |
| Q4 | 3130(9.7) | 111(3.5) | 3019(96.5) | 328(10.5) | 2802(89.5) |
| Q5- Relative greater access to economic resources | 2271(7.0) | 71(3.1) | 2200(96.9) | 200(8.8) | 2071(91.2) |
| **SEIFA Index of Education and Occupation^1^** |  |  |  |  |  |
| Q1- Lower education and occupation status | 8306(25.7) | 321(3.9) | 7985(96.1) | 810(9.8) | 7496(90.2) |
| Q2 | 4829(14.9) | 167(3.5) | 4662(96.5) | 456(9.4) | 4373(90.6) |
| Q3 | 5624(17.4) | 244(4.3) | 5380(95.7) | 584(10.4) | 5040(89.6) |
| Q4 | 7285(22.5) | 278(3.8) | 7007(96.2) | 725(10.0) | 6560(90.0) |
| Q5- Higher education and occupation status | 6251(19.3) | 197(3.2) | 6054(96.8) | 578(9.2) | 5673(90.8) |
| **Marital status** |  |  |  |  |  |
| Single | 1685(5.2) | 65(3.9) | 1620(96.1) | 146(8.7) | 1539(91.3) |
| Married | 11967(37.0) | 465(3.9) | 11502(96.1) | 1118(9.3) | 10849(90.7) |
| Widowed | 15839(49.0) | 580(3.7) | 15259(96.3) | 1634(10.3) | 14205(89.7) |
| Divorced, separated or not determined | 2825(8.7) | 99(3.5) | 2726(96.5) | 258(9.1) | 2567(90.9) |
| **Health conditions reported at assessments^2^** |  |  |  |  |  |
| Abnormalities of gait and mobility | 3696(11.4) | 117(3.2) | 3579(96.8) | 323(8.7) | 3373(91.3) |
| Acute and chronic ischaemic heart disease | 4541(14.1) | 182(4.0) | 4359(96.0) | 477(10.5) | 4064(89.5) |
| Amnesia | 4687(14.5) | 198(4.2) | 4489(95.8) | 522(11.1) | 4165(88.9) |
| Anaemia | 1845(5.7) | 78(4.2) | 1767(95.8) | 177(9.6) | 1668(90.4) |
| Back problems—dorsopathies | 3444(10.7) | 110(3.2) | 3334(96.8) | 342(9.9) | 3102(90.1) |
| Blindness | 1613(5.0) | 54(3.3) | 1559(96.7) | 155(9.6) | 1458(90.4) |
| Bowel/faecal incontinence | 1815(5.6) | 75(4.1) | 1740(95.9) | 166(9.1) | 1649(90.9) |
| Cancers | 6949(21.5) | 243(3.5) | 6706(96.5) | 567(8.2) | 6382(91.8) |
| Other neoplasms (includes benign tumours and tumours of uncertain/unknown behaviour) | 701(2.2) | 26(3.7) | 675(96.3) | 58(8.3) | 643(91.7) |
| Cataracts | 1840(5.7) | 55(3.0) | 1785(97.0) | 176(9.6) | 1664(90.4) |
| Cerebrovascular diseases | 6072(18.8) | 233(3.8) | 5839(96.2) | 557(9.2) | 5515(90.8) |
| Chronic lower respiratory diseases | 6263(19.4) | 242(3.9) | 6021(96.1) | 598(9.5) | 5665(90.5) |
| Congestive heart failure | 3835(11.9) | 172(4.5) | 3663(95.5) | 348(9.1) | 3487(90.9) |
| Deafness/hearing loss | 4935(15.3) | 187(3.8) | 4748(96.2) | 518(10.5) | 4417(89.5) |
| Depression, mood and affective disorders, bipolar | 13717(42.4) | 564(4.1) | 13153(95.9) | 1402(10.2) | 12315(89.8) |
| Diabetes | 7761(24.0) | 303(3.9) | 7458(96.1) | 753(9.7) | 7008(90.3) |
| Diseases of the intestine | 4757(14.7) | 180(3.8) | 4577(96.2) | 462(9.7) | 4295(90.3) |
| Disorders of the thyroid gland | 2852(8.8) | 111(3.9) | 2741(96.1) | 310(10.9) | 2542(89.1) |
| Disorientation | 2421(7.5) | 107(4.4) | 2314(95.6) | 272(11.2) | 2149(88.8) |
| Glaucoma | 2325(7.2) | 93(4.0) | 2232(96.0) | 230(9.9) | 2095(90.1) |
| Has other health condition not elsewhere specified | 3066(9.5) | 126(4.1) | 2940(95.9) | 275(9.0) | 2791(91.0) |
| Heart disease | 6262(19.4) | 276(4.4) | 5986(95.6) | 671(10.7) | 5591(89.3) |
| High cholesterol | 5268(16.3) | 183(3.5) | 5085(96.5) | 515(9.8) | 4753(90.2) |
| Hypertension | 16886(52.3) | 600(3.6) | 16286(96.4) | 1679(9.9) | 15207(90.1) |
| Kidney and urinary system disorders | 3455(10.7) | 137(4.0) | 3318(96.0) | 345(10.0) | 3110(90.0) |
| Malaise and fatigue | 2025(6.3) | 76(3.8) | 1949(96.2) | 161(8.0) | 1864(92.0) |
| Myocardial infarction | 1232(3.8) | 55(4.5) | 1177(95.5) | 123(10.0) | 1109(90.0) |
| Neurotic, stress-related & somatoform disorders | 5678(17.6) | 209(3.7) | 5469(96.3) | 525(9.2) | 5153(90.8) |
| Oedema not elsewhere classified | 2725(8.4) | 106(3.9) | 2619(96.1) | 285(10.5) | 2440(89.5) |
| Other arthritis and related disorders | 19199(59.4) | 717(3.7) | 18482(96.3) | 2008(10.5) | 17191(89.5) |
| Other diseases of the circulatory system | 3249(10.1) | 131(4.0) | 3118(96.0) | 325(10.0) | 2924(90.0) |
| Other diseases of the digestive system | 4793(14.8) | 188(3.9) | 4605(96.1) | 484(10.1) | 4309(89.9) |
| Other diseases of the genitourinary system | 2012(6.2) | 83(4.1) | 1929(95.9) | 197(9.8) | 1815(90.2) |
| Other diseases of the nervous system | 2163(6.7) | 70(3.2) | 2093(96.8) | 214(9.9) | 1949(90.1) |
| Other diseases of the skin and subcutaneous tissue | 1718(5.3) | 65(3.8) | 1653(96.2) | 158(9.2) | 1560(90.8) |
| Other diseases of upper respiratory tract | 216(0.7) | <6 | <6 | 18(8.3) | 198(91.7) |
| Other disorders of the musculoskeletal system & connective tissue | 2281(7.1) | 73(3.2) | 2208(96.8) | 235(10.3) | 2046(89.7) |
| Other heart diseases | 4111(12.7) | 166(4.0) | 3945(96.0) | 424(10.3) | 3687(89.7) |
| Pain | 5996(18.6) | 207(3.5) | 5789(96.5) | 593(9.9) | 5403(90.1) |
| Parkinson’s disease | 1830(5.7) | 70(3.8) | 1760(96.2) | 192(10.5) | 1638(89.5) |
| Poor vision (low vision one or both eyes, unspecified visual loss) | 2895(9.0) | 107(3.7) | 2788(96.3) | 287(9.9) | 2608(90.1) |
| Incontinence (urinary or bowel) | 9347(28.9) | 364(3.9) | 8983(96.1) | 981(10.5) | 8366(89.5) |
| Transient cerebral ischaemic attacks | 1825(5.6) | 75(4.1) | 1750(95.9) | 194(10.6) | 1631(89.4) |
| Urinary tract infection | 2193(6.8) | 84(3.8) | 2109(96.2) | 234(10.7) | 1959(89.3) |
| **Type of Provider** |  |  |  |  |  |
| Not for profit | 20207(62.5) | 749(3.7) | 19458(96.3) | 2014(10.0) | 18193(90.0) |
| For profit | 10006(31.0) | 396(4.0) | 9610(96.0) | 970(9.7) | 9036(90.3) |
| Government | 2103(6.5) | 64(3.0) | 2039(97.0) | 172(8.2) | 1931(91.8) |

IQR=Interquartile range. SEIFA=Socio-Economic Indexes for Areas. ROSA=Registry of Senior Australians.

1. Missing data N(%):English the first language 52(0.2), Born in Australia 102(0.3), ROSA Frailty index score 65(0.2).
2. Conditions were ascertained using the aged care eligibility or entry into care assessments. Only conditions with a >0.1% prevalence are shown. Certain conditions with multiple subgroups were possible are combined for reporting (i.e. diabetes, dementia, cerebrovascular disease, cardiovascular disease, cancer).

**Supplementary Table 3.** ***Individual characteristics* at entry into permanent residential aged care from Aged Care Funding Instrument Assessment^1^ by fall-related hospitalisations within 90 days or 365 days of entry (not included in Table 1)**

| **Characteristics** | **Total N(%)** | **Fall within 90 days N(%)** | **No fall within 90 days N(%)** | **Fall within 365 days N(%)** | **No fall within 365 days N(%)** |
| --- | --- | --- | --- | --- | --- |
| **Total^1^** | 32316 | 1209(3.7) | 31107(96.3) | 3156(9.8) | 29160(90.2) |
| **Nutrition rating** |  |  |  |  |  |
| A best | 3399(10.5) | 67(2.0) | 3332(98.0) | 267(7.9) | 3132(92.1) |
| B | 7197(22.3) | 187(2.6) | 7010(97.4) | 641(8.9) | 6556(91.1) |
| C | 15325(47.4) | 665(4.3) | 14660(95.7) | 1766(11.5) | 13559(88.5) |
| D worst | 4950(15.3) | 224(4.5) | 4726(95.5) | 344(6.9) | 4606(93.1) |
| **Mobility rating** |  |  |  |  |  |
| A best | 1095(3.4) | 16(1.5) | 1079(98.5) | 61(5.6) | 1034(94.4) |
| B | 2290(7.1) | 45(2.0) | 2245(98.0) | 192(8.4) | 2098(91.6) |
| C | 12846(39.8) | 370(2.9) | 12476(97.1) | 1378(10.7) | 11468(89.3) |
| D worst | 14640(45.3) | 712(4.9) | 13928(95.1) | 1387(9.5) | 13253(90.5) |
| **Hygiene rating** |  |  |  |  |  |
| A best | 109(0.3) | <6 | <6 | 6(5.5) | 103(94.5) |
| B | 2787(8.6) | 41(1.5) | 2746(98.5) | 210(7.5) | 2577(92.5) |
| C | 5204(16.1) | 127(2.4) | 5077(97.6) | 494(9.5) | 4710(90.5) |
| D worst | 22771(70.5) | 972(4.3) | 21799(95.7) | 2308(10.1) | 20463(89.9) |
| **Toileting rating** |  |  |  |  |  |
| A best | 1484(4.6) | 25(1.7) | 1459(98.3) | 105(7.1) | 1379(92.9) |
| B | 5192(16.1) | 94(1.8) | 5098(98.2) | 428(8.2) | 4764(91.8) |
| C | 5734(17.7) | 184(3.2) | 5550(96.8) | 628(11.0) | 5106(89.0) |
| D worst | 18461(57.1) | 840(4.6) | 17621(95.4) | 1857(10.1) | 16604(89.9) |
| **Continence rating** |  |  |  |  |  |
| A best | 6875(21.3) | 154(2.2) | 6721(97.8) | 573(8.3) | 6302(91.7) |
| B | 1838(5.7) | 49(2.7) | 1789(97.3) | 161(8.8) | 1677(91.2) |
| C | 1906(5.9) | 54(2.8) | 1852(97.2) | 186(9.8) | 1720(90.2) |
| D worst | 20252(62.7) | 886(4.4) | 19366(95.6) | 2098(10.4) | 18154(89.6) |
| **Cognitive rating^2^** |  |  |  |  |  |
| A best | 4617(14.3) | 97(2.1) | 4520(97.9) | 290(6.3) | 4327(93.7) |
| B | 9742(30.1) | 297(3.0) | 9445(97.0) | 902(9.3) | 8840(90.7) |
| C | 9677(29.9) | 384(4.0) | 9293(96.0) | 1034(10.7) | 8643(89.3) |
| D worst | 6835(21.2) | 365(5.3) | 6470(94.7) | 792(11.6) | 6043(88.4) |
| **Wandering rating** |  |  |  |  |  |
| A best | 23649(73.2) | 873(3.7) | 22776(96.3) | 2196(9.3) | 21453(90.7) |
| B | 1975(6.1) | 85(4.3) | 1890(95.7) | 222(11.2) | 1753(88.8) |
| C | 1268(3.9) | 42(3.3) | 1226(96.7) | 150(11.8) | 1118(88.2) |
| D worst | 3979(12.3) | 143(3.6) | 3836(96.4) | 450(11.3) | 3529(88.7) |
| **Verbal behaviour rating** |  |  |  |  |  |
| A best | 7672(23.7) | 238(3.1) | 7434(96.9) | 661(8.6) | 7011(91.4) |
| B | 4395(13.6) | 162(3.7) | 4233(96.3) | 442(10.1) | 3953(89.9) |
| C | 4689(14.5) | 165(3.5) | 4524(96.5) | 469(10.0) | 4220(90.0) |
| D worst | 14115(43.7) | 578(4.1) | 13537(95.9) | 1446(10.2) | 12669(89.8) |
| **Physical behaviour rating** |  |  |  |  |  |
| A best | 15124(46.8) | 462(3.1) | 14662(96.9) | 1391(9.2) | 13733(90.8) |
| B | 3884(12.0) | 162(4.2) | 3722(95.8) | 404(10.4) | 3480(89.6) |
| C | 3037(9.4) | 117(3.9) | 2920(96.1) | 316(10.4) | 2721(89.6) |
| D worst | 8826(27.3) | 402(4.6) | 8424(95.4) | 907(10.3) | 7919(89.7) |
| **Depression and dysthymia rating^3^** |  |  |  |  |  |
| A best | 13872(42.9) | 457(3.3) | 13415(96.7) | 1261(9.1) | 12611(90.9) |
| B | 9246(28.6) | 341(3.7) | 8905(96.3) | 923(10.0) | 8323(90.0) |
| C | 4353(13.5) | 205(4.7) | 4148(95.3) | 490(11.3) | 3863(88.7) |
| D worst | 3400(10.5) | 140(4.1) | 3260(95.9) | 344(10.1) | 3056(89.9) |
| **Medication assistance rating** |  |  |  |  |  |
| A best | 522(1.6) | <6 | <6 | 32(6.1) | 490(93.9) |
| B | 16627(51.5) | 577(3.5) | 16050(96.5) | 1696(10.2) | 14931(89.8) |
| C | 7412(22.9) | 293(4.0) | 7119(96.0) | 727(9.8) | 6685(90.2) |
| D worst | 6310(19.5) | 268(4.2) | 6042(95.8) | 563(8.9) | 5747(91.1) |
| **Complex health care rating** |  |  |  |  |  |
| A best | 3100(9.6) | 75(2.4) | 3025(97.6) | 239(7.7) | 2861(92.3) |
| B | 7284(22.5) | 222(3.0) | 7062(97.0) | 678(9.3) | 6606(90.7) |
| C | 9494(29.4) | 352(3.7) | 9142(96.3) | 1033(10.9) | 8461(89.1) |
| D worst | 10993(34.0) | 494(4.5) | 10499(95.5) | 1068(9.7) | 9925(90.3) |

1. Missing data N(%): 1445(4.5) for the whole dataset.
2. Mapped to the Psychogeriatric Assessment Scales (PAS).
3. Mapped to the Cornell Scale for Depression.

**Supplementary Table 4. *Medication use c*haracteristics within 90 days prior to entry into permanent residential aged care by fall-related hospitalisations within 90 days or 365 days of entry (not included in Table 1)**

| **Medication variables** | **Total N(%)** | **Fall within 90 days N(%)** | **No fall within 90 days N(%)** | **Fall within 365 days N(%)** | **No fall within 365 days N(%)** |
| --- | --- | --- | --- | --- | --- |
| **Total** | 32316 | 1209(3.7) | 31107(96.3) | 3156(9.8) | 29160(90.2) |
| **Medications Sedative Load Rating** |  |  |  |  |  |
| 0 | 7764(24.0) | 233(3.0) | 7531(97.0) | 681(8.8) | 7083(91.2) |
| 1-2 | 3579(11.1) | 118(3.3) | 3461(96.7) | 313(8.7) | 3266(91.3) |
| 3+ | 20973(64.9) | 858(4.1) | 20115(95.9) | 2162(10.3) | 18811(89.7) |
| **Specific medications (ATC codes)^1^** |  |  |  |  |  |
| Proton pump inhibitors (A02BC*) | 15231(47.1) | 611(4.0) | 14620(96.0) | 1495(9.8) | 13736(90.2) |
| Propulsives (A03FA*) | 3586(11.1) | 129(3.6) | 3457(96.4) | 279(7.8) | 3307(92.2) |
| Other antiemetics (A04AD*) | 2439(7.5) | 81(3.3) | 2358(96.7) | 225(9.2) | 2214(90.8) |
| Osmotically acting laxatives (A06AD*) | 6721(20.8) | 266(4.0) | 6455(96.0) | 710(10.6) | 6011(89.4) |
| Antipropulsives (A07DA*) | 1840(5.7) | 78(4.2) | 1762(95.8) | 176(9.6) | 1664(90.4) |
| Biguanides (A10BA*) | 3973(12.3) | 150(3.8) | 3823(96.2) | 366(9.2) | 3607(90.8) |
| Sulfonylureas (A10BB*) | 2829(8.8) | 123(4.3) | 2706(95.7) | 286(10.1) | 2543(89.9) |
| Heparin group (B01AB*) | 2878(8.9) | 104(3.6) | 2774(96.4) | 251(8.7) | 2627(91.3) |
| Platelet aggregation inhibitors excl. heparin (B01AC*) | 13113(40.6) | 534(4.1) | 12579(95.9) | 1361(10.4) | 11752(89.6) |
| Iron in combination with folic acid (B03AD*) | 2021(6.3) | 80(4.0) | 1941(96.0) | 210(10.4) | 1811(89.6) |
| Vitamin B12 (cyanocobalamin and analogues) (B03BA*) | 2914(9.0) | 130(4.5) | 2784(95.5) | 311(10.7) | 2603(89.3) |
| Digitalis glycosides (C01AA*) | 3464(10.7) | 180(5.2) | 3284(94.8) | 369(10.7) | 3095(89.3) |
| Organic nitrates (C01DA*) | 4578(14.2) | 192(4.2) | 4386(95.8) | 484(10.6) | 4094(89.4) |
| Aldosterone antagonists (C03DA*) | 2628(8.1) | 117(4.5) | 2511(95.5) | 232(8.8) | 2396(91.2) |
| Beta blocking agents, selective (C07AB*) | 9187(28.4) | 372(4.0) | 8815(96.0) | 956(10.4) | 8231(89.6) |
| Dihydropyridine derivatives (C08CA*) | 6538(20.2) | 258(3.9) | 6280(96.1) | 663(10.1) | 5875(89.9) |
| ACE inhibitors, plain (C09AA*) | 9817(30.4) | 355(3.6) | 9462(96.4) | 944(9.6) | 8873(90.4) |
| Angiotensin II antagonists, plain (C09CA*) | 6658(20.6) | 262(3.9) | 6396(96.1) | 712(10.7) | 5946(89.3) |
| Angiotensin II antagonists and diuretics (C09DA*) | 2643(8.2) | 84(3.2) | 2559(96.8) | 247(9.3) | 2396(90.7) |
| HMG CoA reductase inhibitors (C10AA*) | 14765(45.7) | 538(3.6) | 14227(96.4) | 1445(9.8) | 13320(90.2) |
| Corticosteroids, potent (group III) (D07AC*) | 5854(18.1) | 233(4.0) | 5621(96.0) | 604(10.3) | 5250(89.7) |
| Urinary antispasmodics (G04BD*) | 1922(5.9) | 77(4.0) | 1845(96.0) | 199(10.4) | 1723(89.6) |
| Glucocorticoids (H02AB*) | 5244(16.2) | 205(3.9) | 5039(96.1) | 484(9.2) | 4760(90.8) |
| Thyroid hormones (H03AA*) | 4347(13.5) | 181(4.2) | 4166(95.8) | 465(10.7) | 3882(89.3) |
| Tetracyclines (J01AA*) | 2437(7.5) | 105(4.3) | 2332(95.7) | 242(9.9) | 2195(90.1) |
| Penicillins with extended spectrum (J01CA*) | 4453(13.8) | 194(4.4) | 4259(95.6) | 446(10.0) | 4007(90.0) |
| Beta-lactamase resistant penicillins (J01CF*) | 2021(6.3) | 94(4.7) | 1927(95.3) | 225(11.1) | 1796(88.9) |
| Combinations of penicillins, incl. beta-lactamase inhibitors (J01CR*) | 5951(18.4) | 250(4.2) | 5701(95.8) | 587(9.9) | 5364(90.1) |
| First-generation cephalosporins (J01DB*) | 9993(30.9) | 396(4.0) | 9597(96.0) | 1037(10.4) | 8956(89.6) |
| Trimethoprim and derivatives (J01EA*) | 5520(17.1) | 223(4.0) | 5297(96.0) | 563(10.2) | 4957(89.8) |
| Macrolides (J01FA*) | 3768(11.7) | 131(3.5) | 3637(96.5) | 339(9.0) | 3429(91.0) |
| Fluoroquinolones (J01MA*) | 2305(7.1) | 92(4.0) | 2213(96.0) | 225(9.8) | 2080(90.2) |
| Nucleosides, nucleotides excl. reverse transcriptase inhibitors (J05AB*) | 426(1.3) | 15(3.5) | 411(96.5) | 38(8.9) | 388(91.1) |
| Oxicams (M01AC*) | 1867(5.8) | 64(3.4) | 1803(96.6) | 164(8.8) | 1703(91.2) |
| Preparations inhibiting uric acid production (M04AA*) | 2044(6.3) | 70(3.4) | 1974(96.6) | 183(9.0) | 1861(91.0) |
| Bisphosphonates (M05BA*) | 3609(11.2) | 159(4.4) | 3450(95.6) | 414(11.5) | 3195(88.5) |
| Bisphosphonates, combinations (M05BB*) | 2900(9.0) | 126(4.3) | 2774(95.7) | 338(11.7) | 2562(88.3) |
| Other drugs affecting bone structure and mineralization (M05BX*) | 1818(5.6) | 86(4.7) | 1732(95.3) | 245(13.5) | 1573(86.5) |
| Natural opium alkaloids (N02AA*) | 6761(20.9) | 297(4.4) | 6464(95.6) | 699(10.3) | 6062(89.7) |
| Phenylpiperidine derivatives (N02AB*) | 1630(5.0) | 65(4.0) | 1565(96.0) | 149(9.1) | 1481(90.9) |
| Oripavine derivatives (N02AE*) | 3121(9.7) | 137(4.4) | 2984(95.6) | 313(10.0) | 2808(90.0) |
| Opioids in combination with non-opioid analgesics (N02AJ*) | 4127(12.8) | 168(4.1) | 3959(95.9) | 413(10.0) | 3714(90.0) |
| Other opioids (N02AX*) | 2366(7.3) | 78(3.3) | 2288(96.7) | 216(9.1) | 2150(90.9) |
| Anilides (N02BE*) | 18387(56.9) | 719(3.9) | 17668(96.1) | 1902(10.3) | 16485(89.7) |
| Dopa and dopa derivatives (N04BA*) | 1859(5.8) | 72(3.9) | 1787(96.1) | 193(10.4) | 1666(89.6) |
| Other antipsychotics (N05AX*) | 3549(11.0) | 169(4.8) | 3380(95.2) | 389(11.0) | 3160(89.0) |
| Benzodiazepine derivatives (N05BA*) | 5954(18.4) | 261(4.4) | 5693(95.6) | 656(11.0) | 5298(89.0) |
| Benzodiazepine derivatives (N05CD*) | 6810(21.1) | 285(4.2) | 6525(95.8) | 696(10.2) | 6114(89.8) |
| Non-selective monoamine reuptake inhibitors (N06AA*) | 3395(10.5) | 122(3.6) | 3273(96.4) | 322(9.5) | 3073(90.5) |
| Other antidepressants (N06AX*) | 4603(14.2) | 185(4.0) | 4418(96.0) | 435(9.5) | 4168(90.5) |
| Anticholinesterases (N06DA*) | 4330(13.4) | 176(4.1) | 4154(95.9) | 439(10.1) | 3891(89.9) |
| Selective beta-2-adrenoreceptor agonists (R03AC*) | 5671(17.5) | 217(3.8) | 5454(96.2) | 547(9.6) | 5124(90.4) |
| Adrenergic in combination with corticosteroids or other drugs, excl. anticholinergics (R03AK*) | 4519(14.0) | 182(4.0) | 4337(96.0) | 464(10.3) | 4055(89.7) |
| Anticholinergics (R03BB*) | 3841(11.9) | 150(3.9) | 3691(96.1) | 358(9.3) | 3483(90.7) |
| Antibiotics (S01AA*) | 3241(10.0) | 111(3.4) | 3130(96.6) | 302(9.3) | 2939(90.7) |
| Prostaglandin analogues (S01EE*) | 3049(9.4) | 117(3.8) | 2932(96.2) | 301(9.9) | 2748(90.1) |
| Other ophthalmological (S01XA*) | 4800(14.9) | 215(4.5) | 4585(95.5) | 503(10.5) | 4297(89.5) |

ATC= Anatomical, Therapeutic and Chemical classification codes. Only showing medications with >0.1% prevalence.

**Supplementary Table 5. Hospital related health care utilisation within one year prior of entry into permanent residential aged care by individuals’ fall-related hospitalisations within 90 days or 365 days of entry (not included in Table 1)**

| **Variable** | **Total N(%)** | **Fall within 90 days N(%)** | **No fall within 90 days N(%)** | **Fall within 365 days N(%)** | **No fall within 365 days N(%)** |
| --- | --- | --- | --- | --- | --- |
| **Total** | 32316 | 1209(3.7) | 31107(96.3) | 3156(9.8) | 29160(90.2) |
| **Number of potentially preventable hospitalisations** |  |  |  |  |  |
| None | 27016(83.6) | 967(3.6) | 26049(96.4) | 2596(9.6) | 24420(90.4) |
| 1 | 3645(11.3) | 169(4.6) | 3476(95.4) | 402(11.0) | 3243(89.0) |
| 2-4 | 1519(4.7) | 71(4.7) | 1448(95.3) | 154(10.1) | 1365(89.9) |
| 5+ | 136(0.4) | <6 | <6 | <6 | <6 |
| **Total length of stay in unplanned hospitalisations** |  |  |  |  |  |
| Not hospitalised | 12820(39.7) | 367(2.9) | 12453(97.1) | 1052(8.2) | 11768(91.8) |
| 1-30 days | 14129(43.7) | 608(4.3) | 13521(95.7) | 1545(10.9) | 12584(89.1) |
| 31-90 days | 4945(15.3) | 218(4.4) | 4727(95.6) | 525(10.6) | 4420(89.4) |
| 91+ days | 422(1.3) | 16(3.8) | 406(96.2) | 34(8.1) | 388(91.9) |
| **Total length of stay in potentially preventable hospitalisations** |  |  |  |  |  |
| Not hospitalised | 27016(83.6) | 967(3.6) | 26049(96.4) | 2596(9.6) | 24420(90.4) |
| 1-30 days | 4607(14.3) | 204(4.4) | 4403(95.6) | 488(10.6) | 4119(89.4) |
| 31-90 days | 666(2.1) | 38(5.7) | 628(94.3) | 72(10.8) | 594(89.2) |
| 91+ days | 27(0.1) | <6 | <6 | <6 | <6 |
| **Days between last unplanned hospitalisation and entry into PRAC** |  |  |  |  |  |
| 1-20 | 7050(21.8) | 297(4.2) | 6753(95.8) | 678(9.6) | 6372(90.4) |
| 21-100 | 8972(27.8) | 401(4.5) | 8571(95.5) | 1022(11.4) | 7950(88.6) |
| 101-150 | 1538(4.8) | 58(3.8) | 1480(96.2) | 153(9.9) | 1385(90.1) |
| 151-365 | 1936(6.0) | 86(4.4) | 1850(95.6) | 251(13.0) | 1685(87.0) |
| 365+ | 12820(39.7) | 367(2.9) | 12453(97.1) | 1052(8.2) | 11768(91.8) |
| **Days between last separation from a potentially preventable hospitalisation and entry into PRAC** |  |  |  |  |  |
| 1-20 | 1234(3.8) | 55(4.5) | 1179(95.5) | 109(8.8) | 1125(91.2) |
| 21-100 | 2183(6.8) | 110(5.0) | 2073(95.0) | 244(11.2) | 1939(88.8) |
| 101-150 | 623(1.9) | 21(3.4) | 602(96.6) | 51(8.2) | 572(91.8) |
| 151-365 | 1260(3.9) | 56(4.4) | 1204(95.6) | 156(12.4) | 1104(87.6) |
| 365+ | 27016(83.6) | 967(3.6) | 26049(96.4) | 2596(9.6) | 24420(90.4) |

PRAC=permanent residential aged care.

**Supplementary Table 6. *Other health care service utilisation* within one year prior of entry into permanent residential aged care by individuals’ fall-related hospitalisations within 90 days or 365 days of entry (not included in Table 1)**

| **MBS Service Description (MBS Group), N** | **Total N(%)** | **Fall within 90 days N(%)** | **No fall within 90 days N(%)** | **Fall within 365 days N(%)** | **No fall within 365 days N(%)** |
| --- | --- | --- | --- | --- | --- |
| **Total** | 32316 | 1209(3.7) | 31107(96.3) | 3156(9.8) | 29160(90.2) |
| **Other non-referred attendances to which no other item applies (A02)** |  |  |  |  |  |
| 0 | 29202(90.4) | 1106(3.8) | 28096(96.2) | 2883(9.9) | 26319(90.1) |
| 1 | 1807(5.6) | 68(3.8) | 1739(96.2) | 154(8.5) | 1653(91.5) |
| 2-4 | 863(2.7) | 27(3.1) | 836(96.9) | 79(9.2) | 784(90.8) |
| 5+ | 375(1.2) | 7(1.9) | 368(98.1) | 37(9.9) | 338(90.1) |
| **Optometrical services (A10)** |  |  |  |  |  |
| 0 | 21811(67.5) | 812(3.7) | 20999(96.3) | 2090(9.6) | 19721(90.4) |
| 1 | 8162(25.3) | 299(3.7) | 7863(96.3) | 812(9.9) | 7350(90.1) |
| 2-4 | 2210(6.8) | 95(4.3) | 2115(95.7) | 245(11.1) | 1965(88.9) |
| 5+ | 64(0.2) | <6 | <6 | 6(9.4) | 58(90.6) |
| **Urgent attendance after hours (A11)** |  |  |  |  |  |
| 0 | 21722(67.2) | 780(3.6) | 20942(96.4) | 2052(9.4) | 19670(90.6) |
| 1 | 6648(20.6) | 247(3.7) | 6401(96.3) | 662(10.0) | 5986(90.0) |
| 2-4 | 3435(10.6) | 165(4.8) | 3270(95.2) | 395(11.5) | 3040(88.5) |
| 5+ | 442(1.4) | 16(3.6) | 426(96.4) | 44(10.0) | 398(90.0) |
| **Health assessments (MBS A14)** |  |  |  |  |  |
| 0 | 22369(69.2) | 815(3.6) | 21554(96.4) | 2079(9.3) | 20290(90.7) |
| 1 | 9229(28.6) | 368(4.0) | 8861(96.0) | 1009(10.9) | 8220(89.1) |
| 2+ | 649(2.0) | 25(3.9) | 624(96.1) | 65(10.0) | 584(90.0) |
| **75+ health assessment (MBS Items: 705/707 only)** |  |  |  |  |  |
| 0 | 25501(78.9) | 915(3.6) | 24586(96.4) | 2365(9.3) | 23136(90.7) |
| 1 | 6401(19.8) | 281(4.4) | 6120(95.6) | 747(11.7) | 5654(88.3) |
| 2+ | 345(1.1) | 12(3.5) | 333(96.5) | 41(11.9) | 304(88.1) |
| **GP management plans, team care arrangements, multidisciplinary care plans (A15)** |  |  |  |  |  |
| 0 | 18041(55.8) | 647(3.6) | 17394(96.4) | 1659(9.2) | 16382(90.8) |
| 1 | 3237(10.0) | 125(3.9) | 3112(96.1) | 334(10.3) | 2903(89.7) |
| 2-4 | 10224(31.6) | 403(3.9) | 9821(96.1) | 1069(10.5) | 9155(89.5) |
| 5+ | 745(2.3) | 33(4.4) | 712(95.6) | 91(12.2) | 654(87.8) |
| **Preparation of a GP management plan (MBS Item 721 only)** |  |  |  |  |  |
| 0 | 24311(75.2) | 900(3.7) | 23411(96.3) | 2324(9.6) | 21987(90.4) |
| 1 | 7897(24.4) | 306(3.9) | 7591(96.1) | 824(10.4) | 7073(89.6) |
| 2+ | 39(0.1) | <6 | <6 | <6 | <6 |
| **Coordination of team care arrangements (MBS Item 723 only)** |  |  |  |  |  |
| 0 | 25259(78.2) | 943(3.7) | 24316(96.3) | 2438(9.7) | 22821(90.3) |
| 1 | 6947(21.5) | 264(3.8) | 6683(96.2) | 711(10.2) | 6236(89.8) |
| 2+ | 41(0.1) | <6 | <6 | <6 | <6 |
| **Medication management reviews (A17)** |  |  |  |  |  |
| 0 | 30857(95.5) | 1147(3.7) | 29710(96.3) | 2997(9.7) | 27860(90.3) |
| 1+ | 1390(4.3) | 61(4.4) | 1329(95.6) | 156(11.2) | 1234(88.8) |
| **GP attendance associated with PIP incentive payments (A18)** |  |  |  |  |  |
| 0 | 30808(95.3) | 1149(3.7) | 29659(96.3) | 3003(9.7) | 27805(90.3) |
| 1 | 1433(4.4) | 58(4.0) | 1375(96.0) | 149(10.4) | 1284(89.6) |
| 2+ | <6 | <6 | <6 | <6 | <6 |
| **GP mental health treatment (A20)** |  |  |  |  |  |
| 0 | 31103(96.2) | 1168(3.8) | 29935(96.2) | 3040(9.8) | 28063(90.2) |
| 1 | 807(2.5) | 31(3.8) | 776(96.2) | 86(10.7) | 721(89.3) |
| 2-4 | 283(0.9) | 8(2.8) | 275(97.2) | 21(7.4) | 262(92.6) |
| 5+ | 54(0.2) | <6 | <6 | 6(11.1) | 48(88.9) |
| **GP after-hours attendances to which no other item applies (A22)** |  |  |  |  |  |
| 0 | 20118(62.3) | 732(3.6) | 19386(96.4) | 1860(9.2) | 18258(90.8) |
| 1 | 6846(21.2) | 253(3.7) | 6593(96.3) | 738(10.8) | 6108(89.2) |
| 2-4 | 4103(12.7) | 167(4.1) | 3936(95.9) | 416(10.1) | 3687(89.9) |
| 5+ | 1180(3.7) | 56(4.7) | 1124(95.3) | 139(11.8) | 1041(88.2) |
| **Other non-referred attendances to which no other item applies (A23)** |  |  |  |  |  |
| 0 | 31212(96.6) | 1172(3.8) | 30040(96.2) | 3047(9.8) | 28165(90.2) |
| 1 | 855(2.6) | 31(3.6) | 824(96.4) | 87(10.2) | 768(89.8) |
| 2-4 | 170(0.5) | <6 | <6 | 19(11.2) | 151(88.8) |
| 5+ | <6 | <6 | <6 | <6 | <6 |
| **Pain and palliative medicine (A24)** |  |  |  |  |  |
| 0 | 31821(98.5) | 1192(3.7) | 30629(96.3) | 3127(9.8) | 28694(90.2) |
| 1 | 155(0.5) | <6 | <6 | 13(8.4) | 142(91.6) |
| 2-4 | 126(0.4) | <6 | <6 | <6 | <6 |
| 5+ | 145(0.4) | 9(6.2) | 136(93.8) | 9(6.2) | 136(93.8) |
| **Geriatric medicine (A28)** |  |  |  |  |  |
| 0 | 30807(95.3) | 1138(3.7) | 29669(96.3) | 2993(9.7) | 27814(90.3) |
| 1 | 1171(3.6) | 59(5.0) | 1112(95.0) | 136(11.6) | 1035(88.4) |
| 2+ | 269(0.8) | 11(4.1) | 258(95.9) | 24(8.9) | 245(91.1) |
| **Allied health (part of chronic disease management) (M03)** |  |  |  |  |  |
| 0 | 21980(68.0) | 769(3.5) | 21211(96.5) | 1992(9.1) | 19988(90.9) |
| 1 | 1628(5.0) | 61(3.7) | 1567(96.3) | 156(9.6) | 1472(90.4) |
| 2-4 | 6063(18.8) | 272(4.5) | 5791(95.5) | 711(11.7) | 5352(88.3) |
| 5+ | 2576(8.0) | 106(4.1) | 2470(95.9) | 294(11.4) | 2282(88.6) |
| **Nurse practitioners’ attendances (M14)** |  |  |  |  |  |
| 0 | 179(0.6) | 7(3.9) | 172(96.1) | 11(6.1) | 168(93.9) |
| 1 | 32068(99.2) | 1201(3.7) | 30867(96.3) | 3142(9.8) | 28926(90.2) |
| 2-4 | 113(0.3) | <6 | <6 | 7(6.2) | 106(93.8) |
| 5+ | <6 | <6 | <6 | <6 | <6 |

MBS=Medicare Benefits Schedule. GP= General Practitioners. PIP=Practice Incentive Program.

**Supplementary Table 7. Variables and coding used to recreate risk factors included in the Fracture Risk Assessment Tool for Community Dwelling Older People (FRAT-up)^2^ and prevalence of risk factor in the study cohort**

| **Variables included** | **Coding^1^** | **N(%)** |
| --- | --- | --- |
| **Total Cohort** |  | **32316 (100)** |
| **Health conditions** |  |  |
| History of falls | ACAT/ACFI 1715 | 6938(21) |
| Urinary incontinence | ACAT 1403, 1708, ACFI Q05_R1 = 4, ATCs G04BD01-G04BD99 | 19993(62) |
| Diabetes | ACAT/ACFI 0402, 0403, 0404, ATCs A10AA01:A10BX99 | 8037(25) |
| Parkinson’s disease | ACAT 0604, 0524, ACFI 0604 | 1834(6) |
| Arthritis | ACAT/ACFI 1301, 1302, 1303 | 19712(61) |
| Stroke | ACAT/ACFI 0910, 0911, 0912, 0913, 0914, 0915, 0605 | 7002(22) |
| Depression | ACAT 0552, ACFI Q10 C or D, ATCs N06A* | 16279(50) |
| Pain | ACAT 1704, ACFI Q12_R4 A or B, ATCs N02A*, N02B* | 25778(80) |
| Dizziness | ACAT/ACFI 1718, 1728 | 1846(6) |
| Vision impairment | ACAT/ACFI 0703, 0704 | 4172(13) |
| Hearing impairment | ACAT/ACFI 0802 | 4935(15) |
| Cognitive impairment | ACAT 050*, ACFI Q06 C or D, ATCs N06DA02, N06DA03, N06DA04, N06DX01 | 18764(58) |
| Gait/mobility abnormality | ACAT/ACFI 1714 | 3696(11) |
| **Functional limitations** |  |  |
| Mobility impairment | ACFI Q02 C or D | 27486(85) |
| Physical disability | ACAT AL_SELF, AL_HEALTH, ACFI ADL_LEVEL M or H | 30766(95) |
| Behavioural problems | ACAT/ACFI 1716, 1717, 1719, 1721, 1722, 1723 | 6592(20) |
| Poor nutrition | ACAT/ACFI 1730, 0405, 0406 | 2397(7) |
| **Medications**^2^ |  |  |
| Number unique medications (Median, IQR) | Count of unique medicines at ATC 7-digit level | 7 (4-10) |
| Antihypertensives | ATCs C02*, C03*, C07*, C08*, C09* | 24550(76) |
| Antiepileptics | ATCs N03* | 2249(7) |
| Sedative load: 0 |  | 7764(24) |
| Sedative load: 1-2 |  | 3579(11) |
| Sedative load: 3+ |  | 20973(65) |

ACAT=aged care eligibility assessment performed by an aged care eligibility assessment team. ACFI=aged care funding instrument. ATC= Anatomical, Therapeutic and Chemical classification codes.

1. Coding based on health conditions and activity limitations reported at aged care eligibility (‘ACAT’), health conditions or specific questions at entry into care assessment (‘ACFI’), or medication ATC code as specified.
2. Medications and sedative load rating based on one year prior to entry into care history of medication use. Based on Registry of Senior Australians specifications in its Outcome Monitoring System^3^.

**Supplementary Table 8. Risk Estimates Using the Fracture Risk Assessment Tool for Community Dwelling Older People (FRAT-up)^2^**

|  | **90 days** | | | **365 days** | | |
| --- | --- | --- | --- | --- | --- | --- |
| **Risk Factors** | **sHR** | **95% CI** | **P-value** | **sHR** | **95% CI** | **P-value** |
| Time trend, years | 1.06 | 1.03-1.09 | <0.001 | 1.08 | 1.06-1.10 | <0.001 |
| Age | 1.03 | 1.02-1.04 | <0.001 | 1.02 | 1.02-1.03 | <0.001 |
| Men | 1.19 | 1.06-1.34 | 0.003 | 0.98 | 0.91-1.05 | 0.500 |
| **Health conditions** |  |  |  |  |  |  |
| History of falls | 1.51 | 1.33-1.72 | <0.001 | 1.36 | 1.25-1.48 | <0.001 |
| Urinary incontinence | 1.34 | 1.16-1.54 | <0.001 | 1.08 | 0.99-1.18 | 0.081 |
| Diabetes | 1.04 | 0.92-1.17 | 0.510 | 1.04 | 0.95-1.13 | 0.410 |
| Parkinson’s disease | 0.98 | 0.76-1.26 | 0.860 | 1.17 | 1.02-1.35 | 0.024 |
| Arthritis | 0.92 | 0.82-1.04 | 0.190 | 1.08 | 0.99-1.17 | 0.080 |
| Dizziness | 0.95 | 0.72-1.25 | 0.710 | 0.96 | 0.83-1.12 | 0.630 |
| Stroke | 1.06 | 0.91-1.24 | 0.430 | 1.02 | 0.92-1.13 | 0.740 |
| Depression | 1.23 | 1.09-1.38 | 0.001 | 1.12 | 1.05-1.20 | 0.001 |
| Pain | 1.16 | 0.97-1.38 | 0.110 | 1.14 | 1.04-1.26 | 0.007 |
| Vision impairment | 0.93 | 0.77-1.12 | 0.450 | 0.98 | 0.88-1.10 | 0.780 |
| Hearing impairment | 0.99 | 0.84-1.16 | 0.900 | 1.05 | 0.94-1.16 | 0.400 |
| Cognitive impairment | 1.47 | 1.28-1.69 | <0.001 | 1.27 | 1.18-1.37 | <0.001 |
| Gait/mobility abnormality | 0.76 | 0.62-0.93 | 0.007 | 0.82 | 0.72-0.93 | 0.001 |
| **Functional Limitations** |  |  |  |  |  |  |
| Mobility impairment | 1.58 | 1.21-2.05 | 0.001 | 1.11 | 0.97-1.27 | 0.130 |
| Physical disability | 1.27 | 0.80-2.01 | 0.310 | 0.98 | 0.81-1.17 | 0.800 |
| Behavioural problems | 1.14 | 0.99-1.30 | 0.068 | 1.16 | 1.07-1.26 | <0.001 |
| Poor nutrition | 1.11 | 0.91-1.37 | 0.290 | 0.91 | 0.79-1.04 | 0.160 |
| **Medications** |  |  |  |  |  |  |
| Number unique medications (per 1 unit increase) | 1.02 | 1.00-1.03 | 0.051 | 1.00 | 0.99-1.01 | 0.980 |
| Use of antihypertensive | 0.93 | 0.80-1.07 | 0.320 | 1.06 | 0.97-1.16 | 0.230 |
| Sedative load: 1-2 vs 0 | 1.01 | 0.84-1.22 | 0.880 | 1.02 | 0.91-1.16 | 0.710 |
| Sedative load: 3+ vs 0 | 1.22 | 1.04-1.42 | 0.016 | 1.10 | 1.00-1.22 | 0.054 |
| Use of antiepileptic | 0.98 | 0.79-1.21 | 0.830 | 1.04 | 0.90-1.20 | 0.570 |

sHR= sub-distribution hazard ratio. CI=Confidence intervals.

**References**

1. Australian Government, Department of Health and Ageing. Aged Care Funding Instrument. User Guide. (<https://webarchive.nla.gov.au/awa/20191107024615/https://agedcare.health.gov.au/funding/aged-care-subsidies-and-supplements/residential-care-subsidy/basic-subsidy-amount-aged-care-funding-instrument/aged-care-funding-instrument-acfi-user-guide>).

2. Cattelani L, Palumbo P, Palmerini L, et al. FRAT-up, a Web-based fall-risk assessment tool for elderly people living in the community. J Med Internet Res 2015;17(2):e41.

3. Inacio MC, Lang C, Caughey GE, et al. The Registry of Senior Australians outcome monitoring system: quality and safety indicators for residential aged care. Int J Qual Health Care 2020;32(8):502-510.
